# Famous Poisoners in Fiction

**Published:** 1893-08-05

**Authors:** 


### Famous Poisoners in Fiction.

### INTRODUCTORY.

The old-fashioned idea of a novel was a story with a good plot; the new fangled idea is not a story at all, but a character sketch. *Que voulez vous?* In either case, the hand of genius writes of things unreal as if they were real, and the author—if worthy of the name—tells the story of his book people so vividly that they seem to us far more truly alive than are our actual next door neighbours, to whom, may be, we have never even spoken. The poisoner has always played a great part in the history of fiction. He or she makes such a fine villain. There is ever a something mysterious, silent, awful in the thought of poison, besides it is a much more fascinating way than any other of killing off the desired victim; not so vulgar, so disgusting, so revolting as chopping off a head, for instance, as the heroine in Thackeray's Catherine does in her second—and this time fatal—attempt at murder. But poisoning is not confined to the realm of fiction. It is a strange fact, and one which has struck us forcibly whilst arranging this series of articles for The Hospital on the "Famous Poisoners of Fiction," that in nearly every single instance the characters of these vile wretches who with serpentine art, silent, subtle, deliberate, try, for the sake of gain or revenge, to take the God-given life of their fellowmen are founded on fact, *i.e.*, have had their living prototypes suggesting to the author their fictitious counterparts. Lucretia, by Bulwer Lytton, is one instance; Elixir, the poisoner of poisoners, appears, his identity scarce hidden, in many a French novel; the doctor in the "Star Chamber" actually lived, whilst that arch poisoner, most cruel, most horrible of women, the Marquise de Brinvilliers, has served many a time as the heroine for a romance or play, about which people have cried, "Bah! how authors do exaggerate." Do they indeed?

For the sake of gain or revenge we write it is true! It is these two great influences which cause great crimes. For gain, money, the love of which, the Apostle tells us, is the root of all evil, to obtain that gold, bright hard gold, for themselves or their children, men and women have dastardly, cruelly, slain the unsuspecting. For gain or for revenge, that cruellest of passions, that harshest of sins, that most implacable of the demons men harbour in their hearts.

"Revenge is sweet, especially to women," the most sarcastic as well as the most musical of our poets tells us. In regard to poisoners in fiction, it is a strange fact that the greatest number by far are women. Yet not so strange after all. For fiction is real life condensed, and in real life in the majority of cases of poison the poisoners have been women. Yes! those guilty ones who have committed this basest of acts are usually of the gentler sex. We know that the recollection of Neill, Lamson, and others, will arise at once to the mind in contradiction of this statement, but how many cases in a court of law can be cited on the other side. Wholesale husband murders, such as the revelations which horrified the world a few years ago during the murder trials in Hungary; as well as the fact that many and many a fair woman has stood in the felon's dock accused of poisoning during the last half century alone. Another point to notice is that in both the above quoted cases of male poisoners the medical art was represented; indeed, the poisoners amongst men are in nearly every case doctors. Sometimes we wonder if in their case (to quote the words of Shakespeare) "the sight of means to do ill-deeds makes ill-deeds done." But of course there are notable exceptions to this statement with regard to women poisoners, but there is a deep truth in Mr. Charles Yellowplush's explanation. Speaking of Lady Griffins' little arrangements, regarding the "jewel" between De l'Orge and the Hon. Mr. Deuceace, he says, "but it was the *woman* who did that; a man don't deal such fowl blows, igspecially a father to his son; a woman may, poar thing! She's no other means of reventch, and is used to fight with underhand wepns all her life through!" Poor, cruel Lady Griffins! poor, cruel woman! She does not strike an honest blow, it would be unladylike; she therefore has recourse to strategy, to skill in occult arts, to deep planning, to careful and deliberate scheming when on murderous act intent.

In conclusion, we must say one or two words regarding the novels chosen to illustrate this series of articles, of which this preliminary article is a kind of prologue. It has been wished to make them as typical as possible. we know many a reader will exclaim, "Oh, why is such a character not mentioned? Why! does not the writer know of this or that famous novel?" Certainly; but there is an end even to a series of articles, and from an *embarras de richesses* one must choose. Our aim has been to have the characters and scenes as varied as possible. Some of the novels named deal with the middle ages—that dark era when the art of poisoning reached its climax; others, again, deal with the "golden age" of Anne. Others are present day novels, whilst foreign authors, as well as English, will receive our consideration.

The subject is a gruesome one, and if the thoughts these short articles give rise to are sometimes the reverse of pleasant, blame not the writer, but life, and those tiresome authors who will make their fictitious history so sadly true thereunto. The poisoner's is a sorry art, but "'Tis true, 'tis pity; pity 'tis, 'tis true" that men and women—or, perhaps we ought to say, women and men—are, alas! disposed at times to make use of this way of getting rid of troublesome friends and foes—a convenient way for the murderer, whatever the murdered might think of the matter.

### WHEEL OR WOE!

It is strange in this age, claimed to be a nervous one, that inventors should be continually producing new amusements of a nerve trying order. The latest of these terror-producing machines is the great Ferris Wheel at the World's Fair. The wheel is 825 feet in circumference. It is of spider web appearance, practically two wheels being joined together with space between. Fixed between the tyres of the two frames 36 cars are placed at intervals the whole way round it. In these 1,200 passengers can revolve with the wheel, a feat that can be accomplished in seven minutes, though in practice a much longer time is usual. The revolution is performed with remarkable smoothness, but as the greatest height attained is very considerable, such an aeronaut performance is rather more sensational than pleasant to many. The cost of this monster wheel was £80,000; the axle of the wheel is the largest piece of trussed steel ever made.

